# Salt marshes to adapt the flood defences along the Dutch Wadden Sea coast

**DOI:** 10.1007/s11027-015-9640-5

**Published:** 2015-03-25

**Authors:** Jantsje M. van Loon-Steensma

**Affiliations:** Earth System Science Group, Wageningen University & Research Centre, P.O. Box 47, 6700 AA Wageningen, The Netherlands

**Keywords:** Adaptation strategy, Delta Programme, Flood protection, Salt marsh potential map, Wave damping

## Abstract

Concern about the effects of climate change have set in motion a search for flood protection measures to adapt coastlines to the foreseen accelerated sea level rise. In this context, the potential role of salt marshes to adapt the Wadden Sea’s flood defences was explored in the Netherlands Wadden Region Delta Programme. This paper provides an overview of the steps taken by the programme in developing a climate change adaptation strategy so that others might learn from its experiences. The second aim is to summarize the knowledge generated by the programme on the potential role of salt marshes as part of a climate change adaption strategy. Explorative modelling results indicate that Wadden Sea salt marshes affect wave heights, even under extreme conditions. Therefore, a salt-marsh zone in front of the Wadden Sea dikes that could keep pace with sea level rise may result in a reduced dike reinforcement task. A salt marsh potential map gives a rough impression of locations that are potentially interesting for salt marsh conservation and development, based on the current situation, on available information about abiotic conditions for salt marsh formation and the habitats present in the coastal zone. Besides elongated stretches were seminatural salt marshes are already present or developing, several stretches along the Dutch Wadden Sea coast have favourable abiotic conditions for salt marsh development. However, the prospects for integrating salt marshes into flood defences depend also on other aspects. Various nature conservation agreements are in effect with their associated obligations. Furthermore, the foreseen value of salt marsh development compared to traditional reinforcements, in terms of both costs and benefits, must be considered.

## Introduction

### Background

Concern about the effects of climate change (Intergovernmental Panel on Climate Change 2007) has set in motion a search for flood protection measures to adapt coastlines to the foreseen accelerated sea level rise. This certainly applies to the Netherlands, where the state’s Second Delta Committee has recommended increasing flood protection levels and development along with climate change and ecological processes (Deltacommissie 2008). In response to this advice, an integral policy programme, called the Delta Programme, was launched in 2010 with the objective of protecting the Netherlands from flooding and ensuring adequate supplies of freshwater for future generations (Delta Commissioner 2010). As part of this national effort, the Wadden Region Delta Programme has focused on the northern provinces with the aim of developing a long-term strategy to adapt to climate change while also strengthening the Wadden region’s unique nature and landscape values (Ministerie van Verkeer en Waterstaat et al. 2010).

The current paper looks back at the Wadden Region Delta Programme with two objectives. The first is to provide an overview of the steps taken by the programme in developing a climate change adaptation strategy so that others might learn from its experiences. The second objective is to summarize for an international audience the knowledge generated by the programme on the potential role of salt marshes as part of a climate change adaptation strategy, since most of the studies remain in Dutch only.

### Flood protection along the Dutch Wadden Sea coast

**Fig. 1:**
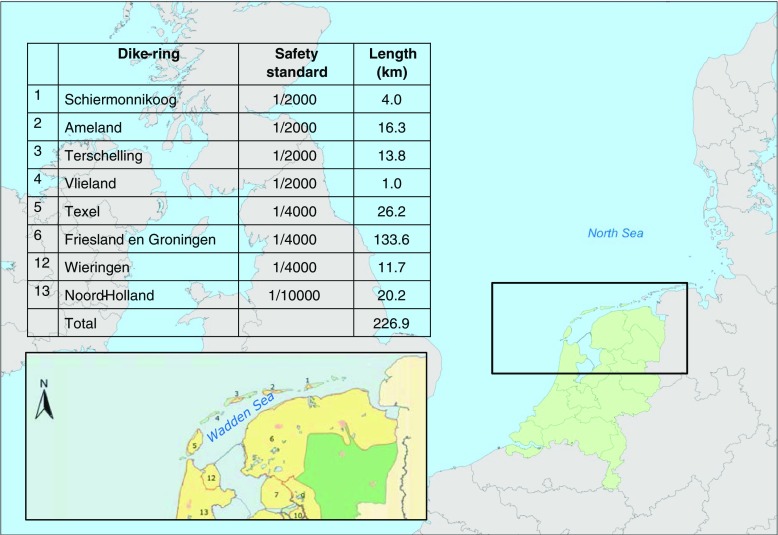
Dike rings in the Dutch Wadden region with safety standard (statistical probability of flooding per number of years) and dike length (km) (Ministerie van Verkeer en Waterstaat 2007b)

Central in the Netherlands Wadden region is the Wadden Sea, one of the world’s largest intertidal areas, renowned for its sand flats and mudflats (see e.g. Wolff 1983; Common Wadden Sea Secretariat (CWSS) 1991; De Jong et al. 1999; Essink et al. 2005; Reise et al. 2010). Furthermore, the Wadden Sea performs a key function in protecting the Dutch mainland from flooding, due to the wave damping capacity of its row of barrier islands and intertidal flats, banks and salt marshes. Some 227 km of dikes protect the Dutch mainland and barrier islands from inundation by the Wadden Sea in addition to the 32-km dike that joins the Dutch provinces of Fryslân and North Holland. These dikes are designed to withstand extreme storm surges with statistical probability of occurring no more than once in 2000 to 10,000 years (Fig. 1). These dikes are constructed with crests well above extreme storm surge levels (~4–5 m + NAP, Ministerie van Verkeer en Waterstaat 2007a) and expected wave run-up. On the northern side of the Dutch barrier islands, facing the North Sea, the primary flood defence system consists of dunes and sandy beaches that are actively maintained by sand nourishments and dune protection programmes. Because the Wadden Sea has a wave damping effect, flood defences along the Wadden Sea coastline are designed for much lower extreme wave heights (~1–2.8 m) than the flood defences (dunes) on the North Sea side of the islands (~10–11 m, Ministerie van Verkeer en Waterstaat 2007a).

Climate change, however, has affected the design requirements of the flood defences, and further shifts are expected in hydraulic conditions (e.g. extreme surge levels, extreme wave heights and wind directions). Climate change could also affect coastal protection by undermining the continued existence of the barrier islands and the extent of the intertidal areas in the Wadden Sea. A supply of sediment into the tidal system is required to keep pace with rising sea levels. If the sediment supply is insufficient, tidal flats and salt marshes may drown (Van Goor et al. 2003), resulting in increased water action on the dikes along the mainland coast. In this context, the Wadden Region Delta Programme initiated studies (i) to assess the effect of climate change on future flood protection, (ii) to develop new flood protection strategies that could boost the ecological resilience of the area while facilitating sustainable human use and (iii) to monitor the effects of climate change on the Wadden Sea system.

Dike designs including vegetated forelands were identified as one promising flood protection strategy for the Wadden region to explore (Deltaprogramma Waddengebied 2011). Already, extensive stretches of salt marsh are found along the coastlines of both the Dutch mainland and the barrier islands. These salt marshes form a vegetated transition zone between land and water. They break incoming waves, reducing wave length and velocity, ultimately dissipating wave energy via friction with vegetation and the marsh surface (e.g. Anderson et al. 2011). Salt marshes thus function as a natural flood defence (see e.g. Brampton 1992; King and Lester 1995; Möller et al. 2001; Costanza et al. 2008; Gedan et al. 2011; Shepard et al. 2011). The action of the marshes in reducing wave height and wave energy could have implications for the required dike dimensions (in particular, dike slope and height) and the need for slope and toe protection structures (e.g. hard revetments and rocks). The presence of salt marshes may have favourable effects on other aspects of dike design as well, such as dike stability and piping (Venema et al. 2012).

Salt marshes, moreover, provide valuable habitat for characteristic salt-marsh vegetation (see e.g. Adam 1990) and for the many migrating wading birds that use the Wadden Sea as a stopover area (e.g. Laursen et al. 2010). In addition to providing a refuge for birds, the salt marshes serve as a spawning area and nursery for fish and provide an important habitat for several invertebrate species (Bakker et al. 2005). In view of these unique biodiversity values, the area is protected by national and international conservation legislation and policies (e.g. the European Union’s (EU) Natura 2000, the EU Water Framework Directive and the Netherlands Spatial Key Decision) (Ministerie van Volkshuisvesting, Ruimtelijke Ordening en Milieu 2007; Ministerie van Verkeer en Waterstaat 2009; Ministerie van Economische Zaken Landbouw en Innovatie 2011), as well as by trilateral agreements between Denmark, Germany and the Netherlands (CWSS 1998; 2010).

From 2011 to 2014, the Wadden Region Delta Programme initiated literature reviews and modelling studies to explore the potential of integrating salt marshes into the Wadden Sea’s flood defence system, including a study of promising locations for implementing this adaptation strategy along the Wadden Sea coast. Furthermore, several stakeholder consultations were organized to deepen insight on the potential for integrating the flood protection service of salt marshes with conservation of nature and landscape values and other functions.

## Steps taken by the Wadden region delta programme to explore the potential of salt marshes in a long-term adaptation strategy

The Wadden Region Delta Programme (henceforth called Wadden Programme) was launched in 2010 as one of six regional sub-programmes of the Dutch Delta Programme (Delta Commissioner 2010). Table 1 summarizes the Wadden Programme’s activities related to the possible inclusion of salt marshes in a long-term climate change adaptation strategy. These activities follow the schedule of the national Dutch Delta Programme, from exploration of possible adaption strategies (2010–2011), to identification and elaboration of promising strategies (2012–2013) and formulation of preferred strategies (2014).

**Table 1:** Steps taken by the Wadden Region Delta Programme to investigate the value of including salt marshes in a long-term climate change adaptation strategy

Year	Phase	Activity	Reports/products
2010	Preparation phase	Formation of the Wadden Region Delta Programme team	
		Exploration of tasks and formulation of objectives of the Wadden Region Delta Programme	*Basisrapport voor het plan van aanpak van het Deltaprogramma Waddengebied* (Oost et al. 2010)
2011	Preliminary exploration of possible adaptation strategies for the Wadden region	Stakeholder consultations to identify and prioritize issues related to long-term climate change adaptation of the Wadden region and definition of research questions	*Quick scans Deltaprogramma Waddengebied* (proposal for eight research topics)
		Explorative literature review	*Een dijk van een kwelder: Een verkenning naar de golfreducerende werking van kwelders* (Van Loon-Steensma et al. 2012a)
		Explorative modelling	*Kwelders en dijkveiligheid in het Waddengebied* (Venema et al. 2012)
2012	Further exploration of future tasks and possible adaptation strategies for the Wadden region	Communication of the results of the explorative studies and consultation among stakeholders concerning possible adaptation strategies	Conference on research findings of the Wadden Region Delta Programme (*Oogstdag Deltaprogramma Waddengebied*)
		Modelling study on future flood protection in the Wadden region	*Toekomstige veiligheidsopgave voor harde keringen in het Waddengebied* (Smale and Hoonhout 2013)
		Literature review on salt-marsh conservation and development techniques	*Stuurbaarheid van Kwelders* (De Groot et al. 2013)
		Identification of promising locations for integrating salt marshes into flood defences in cooperation with stakeholders	*Zoekkaart kwelders en waterveiligheid Waddengebied: een verkenning naar locaties in het Waddengebied waar bestaande kwelders of kwelderontwikkeling mogelijk kunnen bijdragen aan waterveiligheid* (Van Loon-Steensma et al. 2012b)
2013	Identification of promising adaptation strategies for the Wadden region	Communication of the results of the studies and stakeholder consultation on promising adaptation strategies	Conference on the findings of the Wadden Programme (*Oogstdag Deltaprogramma Waddengebied*)
		Stakeholder consultation on management and maintenance of salt marshes	*Eigendom, beheer en gebruik van kwelders in het Waddengebied* (Schrevel and Klostermann 2013)
		Detailed studies on the effects of specific measures	*Doelbereik innovatieve dijkconcepten Deltaprogramma Wadden* (Calderon and Smale 2013)
2014	Defining preferred adaptation strategies for the Wadden region	Summaries of study results of the Wadden Region Delta Programme	*Factsheet betekenis van voorlanden voor waterveiligheid* (Van Loon-Steensma et al. 2014a)
			*Innovatieve dijken als strategie voor een veilig en aantrekkelijk Waddengebied: Samenvatting onderzoek naar innovatieve dijken* (Van Loon-Steensma et al. 2014b)
		Communication of the recommendations of the Wadden Region Delta Programme	Stakeholder meetings
			*Synthesedocument Waddengebied: Achtergronddocument B10* (Gerritsen et al. 2014)

The Wadden Programme began by establishing a coordination group comprised of representatives of national and regional governmental bodies, and local water boards. The coordination group subsequently commissioned research to various experts, starting with an exploration of tasks and objectives and a summary of available knowledge on the impacts of climate change in the Wadden region. In early 2011, interested public and private parties—including inhabitants of the Wadden region—were invited to three meetings to identify and prioritize issues concerning long-term adaptation of the region to climate change and to define pertinent research questions. The possible role of salt marshes for flood protection emerged from this consultation as one of eight topics meriting further investigation. Two explorative studies were then initiated (Table 1). From the first stakeholder meetings, three basic opinions emerged concerning the inclusion of salt marshes in a flood protection strategy: (i) the inclusion of salt marshes in flood defences may offer an opportunity to adapt to the effects of climate change while strengthening the nature and landscape values of the Wadden Sea coast (this view was held by many representatives of local and regional governmental organizations, researchers and inhabitants of the Wadden region); (ii) the inclusion of salt marshes in an adaptation strategy is unpromising because salt marshes do not contribute to flood safety during extreme conditions and, furthermore, creation of salt marshes would substantially increase dike maintenance costs because of the debris they cause on dike grass cover during storm events (this view was expressed by some technical staff members of the water boards); and (iii) inclusion of salt marshes in a flood protection strategy would put at risk the unique nature and biodiversity values of the Wadden Sea (this view was held by some of the Wadden Sea salt-marsh researchers).

The results of both the literature review and the explorative modelling substantiated the potential value of including salt marshes in a climate-change adaptation strategy, but they also identified gaps in the current knowledge. Major gaps concerned (i) the wave damping effect of salt marshes during extreme events, (ii) the potential role of the adjacent intertidal flats in flood protection, (iii) regulations and operational practices needed for incorporating salt marshes in a flood protection strategy for the Netherlands and (iv) localization of promising spots to integrate salt marshes into flood defence systems. In the ensuing years, some aspects of these questions were addressed by studies commissioned by the Wadden Programme (Table 1). Others were taken up by other Dutch research initiatives (e.g. Van Loon-Steensma 2014; Van Wesenbeeck et al. 2014) and in stakeholder consultations. Furthermore, several pilot projects on salt-marsh development were initiated (see e.g. www.ecoshape.nl). Recently, a 4-year research programme was launched to conduct field observations during storm events and to develop methods for assessing how, and how much, vegetated foreshores can contribute to reduced flood risk.

In 2014, integration of salt marshes into flood defences was defined as one of the preferred strategies for the Dutch Wadden region (Gerritsen et al. 2014), leading to its inclusion in a major dikes research programme implemented by the northern water boards. This programme was launched in January 2015 as part of a new national Dutch flood protection programme. Nonetheless, during one of the programme’s inaugural meetings, it became clear that, notwithstanding the research findings and efforts of the Wadden Programme to date, some technical staff of the water boards and a number of Wadden Sea salt-marsh researchers remained resistant to the idea of integrating salt marshes into flood defences.

## Characteristics of the Dutch Wadden Sea salt marshes

### Salt-marsh zones

Salt marshes and their adjacent intertidal flats are a prominent feature of the Wadden Sea (e.g. Wolff 1983; CWSS 1991; De Jong et al. 1999; Essink et al. 2005; Reise et al. 2010). Salt marshes are defined as areas vegetated by salt-tolerant plants and subject to periodic flooding due to the fluctuating water levels of the adjoining saline water body (Adam 1990). They generally develop high in the intertidal zone in sheltered conditions where wave action is limited so that fine sediment can settle and accumulate (Allen and Pye 1992; Allen 2000). Once the upper part of the intertidal zone is no longer submerged with each tide, salt-marsh plants can become established. By trapping sediment, pioneer vegetation contributes to accretion and development of creeks, rendering the environment suitable for species (forbs, grasses and low shrubs) that need more stable sediment and are less tolerant to flooding (in duration as well as frequency) (Adam 1990; Allen 2000). Because of the positive feedback between salt-marsh vegetation and sedimentation, vegetation has an important role in salt-marsh formation (Allen 2000). Salt-marsh plants can thus be understood as eco-engineers, i.e. organisms that physically change the abiotic environment (Jones et al. 1994; Hastings et al. 2007).

Salt-marsh plants are often specialists, restricted to the salt-marsh ecosystem though progressing from a seaward zone of pioneer plant species to more mature plant communities landwards (Adam 1990). The zones represent different stages of vegetation succession. The boundaries of the zones are usually determined by variables like frequency of inundation, sedimentation and erosion, which in turn are related to geological, climatological, vegetation and land use history (Doing 1995; Doody 2008). The floristically most diverse part of the salt marsh is the zone that is submerged regularly but not daily. The highest-elevation zones are flooded only during storm events. Vegetation diversity diminishes with ongoing succession (Dijkema et al. 2001). The vegetated area grades seaward into mudflats or sand flats, from which the vegetated environment is separated by either a ramp or a cliff (Allen and Pye 1992; Allen 1993, 2000). Marshes may experience lateral accretion or erosion. Salt-marsh sediment that erodes during a storm may be redeposited as sand and silt banks.

In salt-marsh zones, the boundary between land and sea shifts with the water level. Like most coastal sedimentary systems, salt-marsh ecosystems are extremely sensitive to changing environmental conditions (Allen 2000). Generally, a moderate sea level rise shapes conditions for marshes to build up by accretion (Allen 2000) or to shift landward. To keep pace with a rising sea level, a permanent supply of sediment into the tidal system is required. If sediment import is insufficient, flats and marshes drown (Van Goor et al. 2003). Most land in the Dutch Wadden region is shielded by dikes, which form a sharp and rigid boundary between land and the dynamic coastal zone. They thus prevent any landward shift of salt-marsh zones, resulting in coastal squeezing (Pontee 2013).

### Salt marshes along the Dutch Wadden Sea coast

**Fig. 2:**
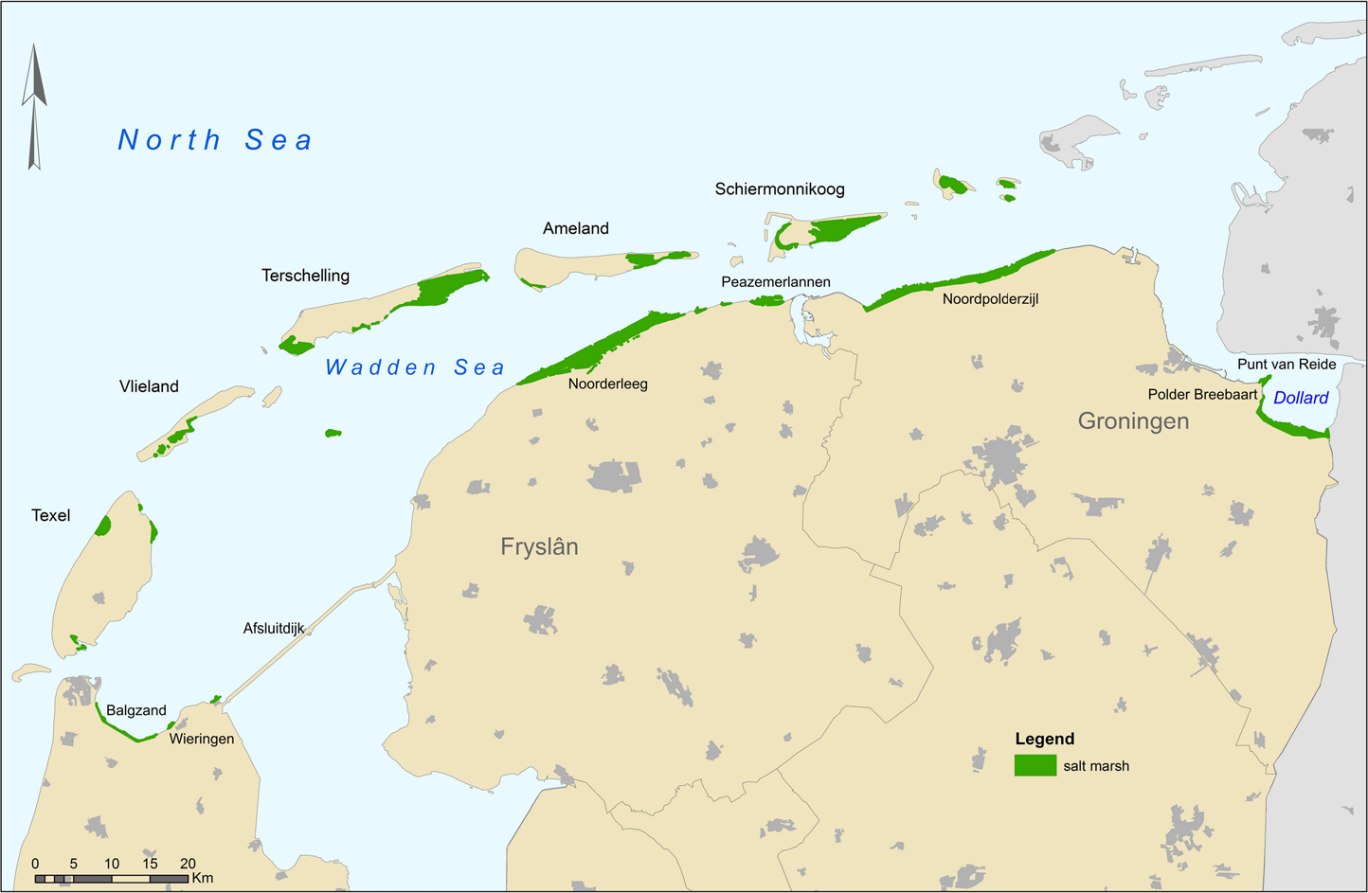
Salt marshes along the Dutch Wadden Sea coast (source: Alterra Habitattypenkaart)

Some 9000 ha of salt marshes are found along the shores of both the Dutch mainland and barrier islands (Dijkema et al. 2008) (Fig. 2). Their elevation in the western part of the Wadden Sea ranges from ~0.6 m + NAP (NAP is the Dutch reference height) on the seaward side to ~1.7 m + NAP on the landward side, and in the Dollard region from ~1.5 m + NAP on the seaward side to ~2.9 m + NAP on the landward side (Actueel Hoogtebestand Nederland (AHN) 2012). The higher elevations from west to east result from differences in tidal range from west to east.

**Fig. 3:**
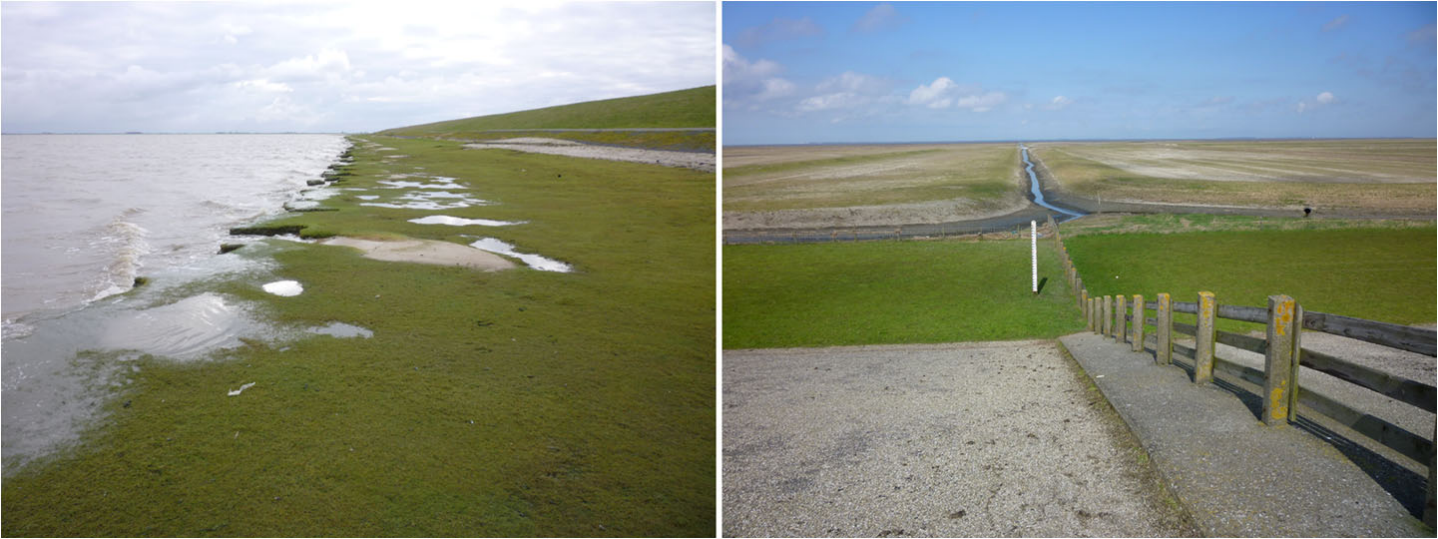
Salt marshes in front of the dike along Polder Breebaart (*left*) and Noorderleeg (*right*)

The marshes along the mainland are mostly the result of constructed accretion works (Dijkema et al. 2013). These still exhibit the historical pattern of rows of drainage ditches situated perpendicular to the coast. On the seaward side, the marshes are protected by sedimentation fields and brushwood dams. Although the salt marshes are the result of human intervention, they nonetheless have many natural features and are highly valued for nature and biodiversity conservation. These salt marshes can therefore be classified as seminatural and have been declared Natura 2000 areas for which target species and protected habitats have been articulated (Ministerie van Economische Zaken, Landbouw en Innovatie 2011). Parts of the salt marshes along the Frisian coast are summer polders that have been either deliberately reconnected to the Wadden Sea by removing parts the summer dike (as in Noorderleeg) or accidentally reconnected after a breach of the low, seaward dike (Paezermerlannen). Along some segments of the dike, the salt-marsh zone is rather narrow (in Wieringen and along the Breebaart Polder dike it is some 10 m), while the salt marshes in North Groningen and the summer polders and salt marshes of Noorderleeg are more than a kilometre wide in some places (Fig. 3).

Prior to the 1970s, in locations with favourable conditions, sedimentation was stimulated by digging drainage systems in the mudflats, planting cordgrass (*Spartina anglica*) and (from the 1930s onwards) constructing brushwood dams (Dijkema et al. 2001). These works aimed at land reclamation. After the 1950s, however, reclamation of salt marshes became economically less feasible, and maintenance of the sedimentation works was reduced or even ceased, initiating a shift towards lateral erosion in various salt-marsh areas (such as Dollard, see e.g. Esselink et al. 2011). Increased awareness of the unique nature and biodiversity values of the Wadden Sea’s salt marshes since the 1970s stimulated a search for best practices to stop the marshes’ steady diminishment (Dijkema et al. 2013). By optimizing the size of the sedimentation fields (the low-lying fields adjacent to the salt marshes), the observed decline was halted, and the salt-marsh area along the Frisian and Groningen coast even increased. Recently, however, the growth of the Frisian salt-marsh area ceased, though the pioneer salt-marsh zone in Groningen is still expanding (Dijkema et al. 2013). Monitoring reveals that during 1960–1995, average accretion rates were 1.8 and 1.2 cm per year, respectively, for the salt marshes along the coast of Fryslân and Groningen (Dijkema et al. 2013). During 1992–2010, accretion rates were, respectively, 1.4 and 1.0 cm per year. Accretion in the Dollard marshes was 0.8 cm per year between 1984 and 2003 (Esselink et al. 2011). Monitoring has also revealed the influence of storm events on accretion of the Wadden Sea salt marshes. The greater frequency of storms since 2000 appears to have accelerated accretion in the higher salt-marsh zone (the zones that are only flooded a few times per year), while accretion in this zone decreased during 1960–2000 because sediment import could not compensate for subsidence (Dijkema et al. 2013). Van Duin et al. (1997) found that at one location (Paezermerlannen), an extreme event with a water level of 2.30 m + NAP resulted in 125 times greater sediment deposition than during a normal tide.

## Flood protection by salt marshes

### Wave damping by salt marshes

Salt marshes form a shallow transition zone from sea to land that attenuates incoming waves. When water depths in this zone diminish to less than the wave base, the wave’s shape is modified and it starts shoaling. Wave length and wave velocity both decrease, and wave height increases before breaking. Wave damping is strongly dependent on the slope of the coastal profile, the water depth above the salt marsh, the width of the salt marsh zone, surface topography and vegetation characteristics (Le Hir et al. 2000; see also studies cited in Anderson et al. 2011 and in Gedan et al. 2011). Wave damping by forelands is determined largely by wave breaking. The exact point at which a wave breaks is a function of wave height (H), water depth (h) and the slope of the coastal profile. The width of a salt marsh hardly affects wave breaking. A broad range of values for H/h have been reported, from 0.45 for a horizontal surface, to 0.60 or even 1.59 for a sloped surface (see Holthuijsen 2007). However, the local topography of forelands tends to be varied, both spatially and temporally. Furthermore, cliff edges (or shallow dams, accretion works, etc.) may influence wave heights locally (Möller et al. 2002).

Wave damping by friction is dependent on the width of the salt marsh and vegetation characteristics (e.g. stem density, plant height, stem diameter and flexibility). Various authors have studied the wave damping capacity of vegetation, in the field as well as in the laboratory (see Tables 1 and 2 in Anderson and Smith 2014). They report wave heights to be significantly more reduced by salt marshes covered with vegetation than by bare sand flats (see e.g. Möller et al. 2001; Yang et al. 2012). Even under simulated storm conditions, 60 % of the observed wave reduction has been attributed to vegetation (Möller et al. 2014). Beyond the presence of vegetation, the effect of several vegetation properties has been investigated as well. For example, zones with a higher plant-stem density of cordgrass (*Spartina alterniflora*) were found to be more effective in wave attenuation than zones with a lower density (Yang et al. 2012). Furthermore, a tall canopy of cordgrass (*Spartina* spp.) in Essex (UK) dampened waves more than a shorter canopy of common glasswort (*Salicornia* spp.) (Möller 2006). Dissipation was roughly three times greater in vegetation with stiff leaves compared to that with flexible leaves (Bouma et al. 2005), but on a biomass basis the dissipation appears to be comparable (Bouma et al. 2010).

**Table 2:** Classes for the potential of salt marsh developing along the Dutch Wadden Sea coast and the defining abiotic parameters (i.e., elevation in relation to tidal range, concentrations of fine-grained sediment and velocity of currents along the coast) (Van Loon-Steensma et al. 2012a, b)

Class	Bathymetry	Concentration fine-grained sediment (%)	Maximum flow velocity (m/s)
Salt marshes already present	>MHWL	>5	<1.2
Natural developing salt marshes	Around MHWL	>5	<1.2
Small efforts required for salt-marsh formation	Around MLWL	>5	<1.2
Larger efforts required for salt marsh formation	Between −5 m NAP and MLWL	>5	>1.2
Unfavourable abiotic conditions for salt-marsh formation	<−5 m NAP	<5	>1.2

*MHWL* mean high water level, *MLWL* mean low water level

### Modelling the effect of Wadden Sea salt marshes on wave heights

As described above, dikes protect the northern provinces of the Netherlands against flooding by the Wadden Sea (Fig. 1). In general, these dikes are soil constructions with a sand core, an outer protective layer of clay and grass (or stone and asphalt), toe protection and a maintenance road. The hydraulic load on the dikes is determined by (i) water level and flow (driven by tidal phase and wind conditions); (ii) wind direction and speed; (iii) North Sea waves, which may penetrate into the Wadden Sea during a storm event; and (iv) local bathymetry and characteristics of the nearshore zone.

The forelands adjacent to the dike thus influence the hydraulic load on the dike and hence may serve to reduce requirements concerning crest height and revetment strength. In order to incorporate the effect of salt marshes on dike design, their effect must be quantified. This has been done using the simulating wave’s nearshore (SWAN) model, which converts wind data into nearshore wave parameters (height, period and direction) for a given location (Booij et al. 1999). The interplay between the various spatial (e.g. topographic) and temporal (e.g. seasonal) factors, however, introduces a significant degree of variability in wave damping that is difficult to model (Van der Meer 2002).

**Fig. 4:**
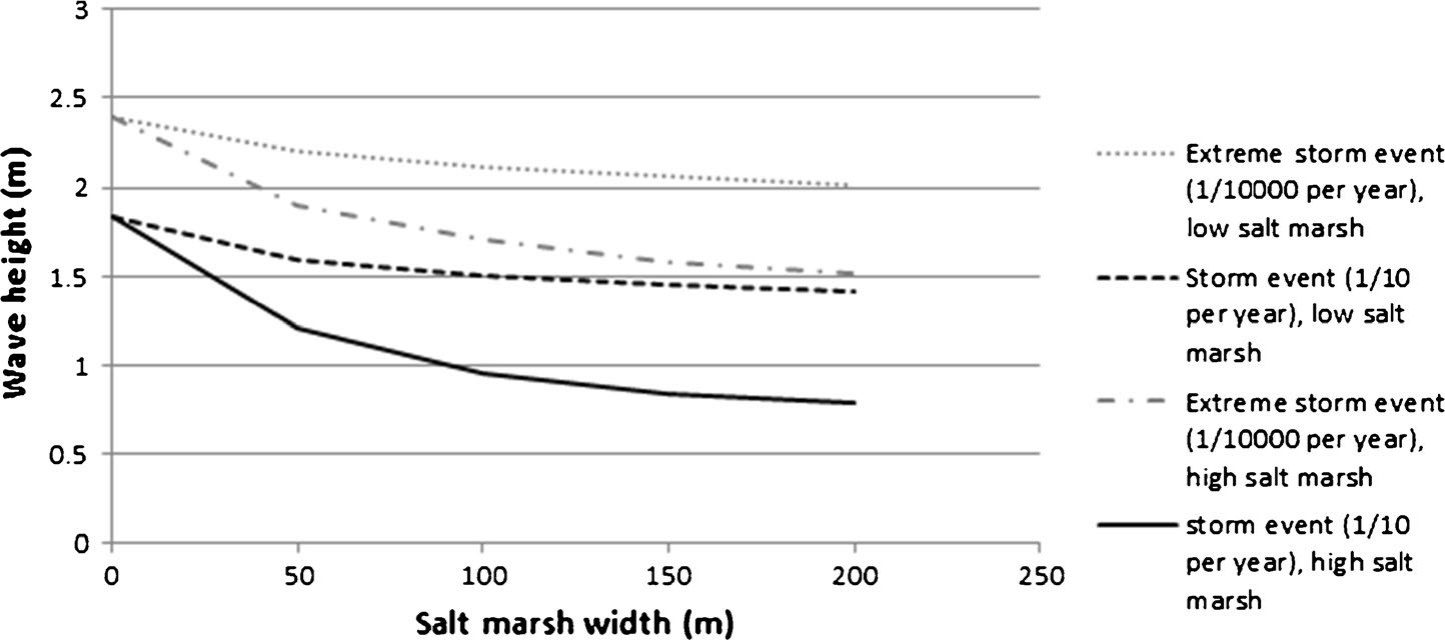
Modelled wave height over a foreshore (1 m + NAP and 2.3 m + NAP) under extreme storm conditions (1/10 and 1/1000 per year) (based on Venema et al. 2012)

To obtain a first impression of the potential of salt marshes for coastal protection in the Wadden region, Venema et al. (2012) modelled wave attenuation over a schematic salt-marsh zone (50–200 m in width, 1.0–2.3 m + NAP in height) under storm and extreme conditions, i.e. with a statistical probability of, respectively, once in 10 years (1/10 per year) and once in 10,000 years (1/10,000 per year). They used information on local storm water levels and wind conditions and assumed a water level and significant wave height of, respectively, 3.6 m + NAP and 1.84 m for a storm (1/10 per year) and 5.0 m + NAP and 2.4 m for an extreme storm event (1/10,000 per year). The results of the modelling work by Venema et al. (2012) (Fig. 4) indicate that even under extreme conditions (1/10,000 per year, with a water level of 5 m + NAP) an elevated foreland (2.3 m + NAP) of 50 m wide dampens waves (2.40 m in height) by some 20 % (resulting in wave heights of 1.90 m). Broadening the foreland to 200 m results in a reduction of 37 % of the initial wave height (resulting in wave heights of 1.50 m). Under storm conditions (1/10 per year), such a wide and high foreland dampens waves (of some 1.84 m) up to 60 % (resulting in wave heights of 0.75 m). However, a low foreland (1 m + NAP) has a modest wave damping effect under extreme conditions (Fig. 4). Under moderate storm conditions, with lower water levels and lower wave heights than in Fig. 4, salt marshes definitely reduce the wave action on the dike. Because such storms occur several times per year, salt marshes would reduce wear and damage on the revetment.

Calderon and Smale (2013) used the SWAN model to investigate the effect of a 600-m salt-marsh zone seaward of all Wadden Sea dikes, in particular on the need for dike reinforcements in the future. As a reference, they employed a schematized standard dike profile meeting current standards. They found that if the salt marshes could keep pace with the rising sea level (0.15 m by 2050), only modest dike reinforcements would be needed in 2050 (heightening approximately 50 km of dikes by <0.25 m). Without a salt-marsh zone, all dikes along the Wadden Sea coast will need to be heightened (by up to 0.5 m). The presence of a salt-marsh zone that can keep pace with a rising sea level would result in unchanged water depth in front of the dike, keeping wave action fairly constant even though sea level may rise. Smale (2014) explored the effect of widening the salt-marsh zone and of the accretion pace. That study found that waves were dampened in the first 1000 m of the salt marsh. Additional width of the salt-marsh zone did not result in more wave damping (Smale 2014). Accretion that outpaces the sea level rise reduces the need for dike reinforcement, again, due to reduced water depth adjacent to the dike. However, salt marshes are present only along certain dike segments in the Wadden Sea (Fig. 2), and some of these marshes are rather narrow. Salt-marsh conservation, development and restoration therefore seem to offer promising avenues to explore for climate change adaptation.

Noteworthy is that salt marshes were already deliberately included in the design of a 12.5-km dike section along the summer polder Noorderleeg. This grass-covered dike was completed in 1992, after flume studies demonstrated that a wide green dike with adjacent polders and salt marshes could withstand extreme storm conditions (Waterloopkundig Laboratorium 1984).

## Salt marsh potential map

**Fig. 5:**
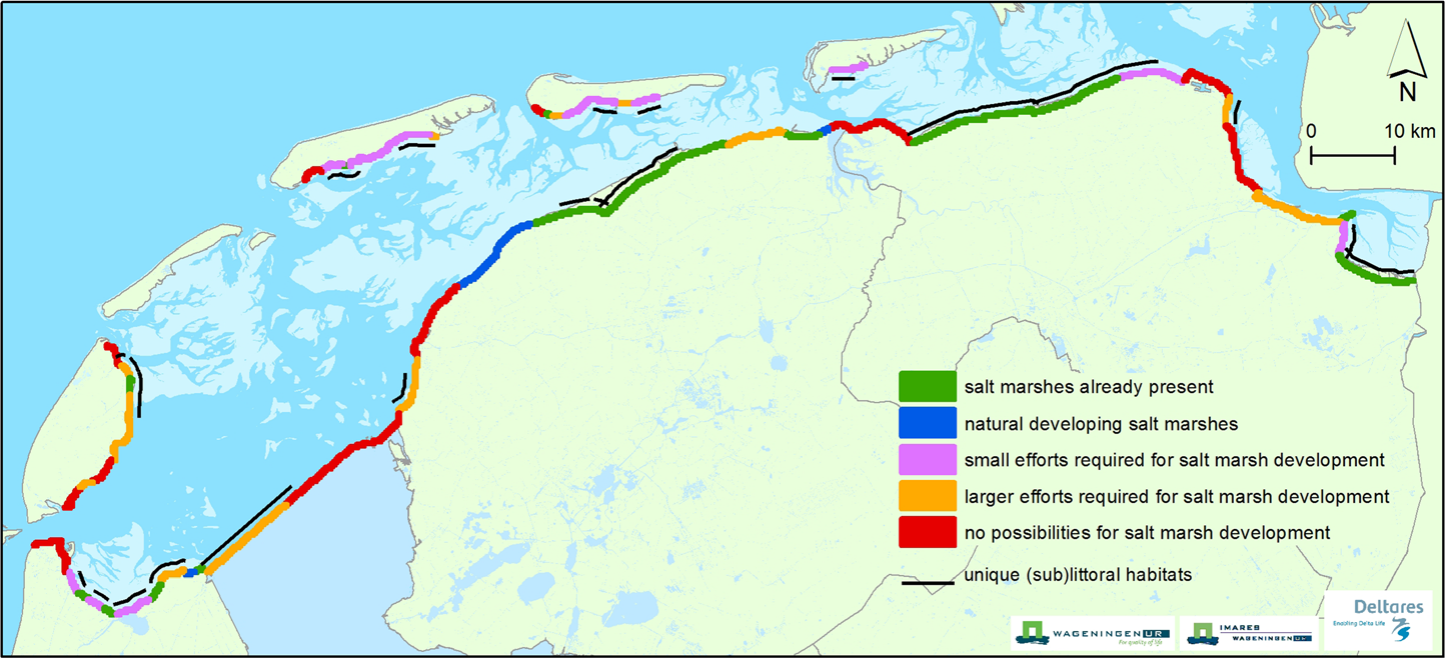
‘Salt marsh potential map’ with locations along the Dutch Wadden Sea coast where (i) seminatural marshes are present, (ii) abiotic conditions are favourable for salt-marsh formation, (iii) salt-marsh development would require minimal effort, (iv) salt-marsh development would require considerable effort, (v) abiotic conditions are considered unsuitable for salt-marsh formation and (vi) unique littoral and sublittoral habitats (Van Loon-Steensma et al. 2012a, b)

The salt marsh potential map (Fig. 5) presents promising locations for integrating salt marshes into the Wadden Sea flood defence system. Identification of these locations is based on the current situation (salt marshes already present, see Fig. 2), an inventory and assessment of the abiotic conditions necessary for salt-marsh formation (Table 2) and biotic characteristics of the coastal zone along the Wadden Sea (both barrier islands and mainland) (Van Loon-Steensma et al. 2012b).

As described in Section 3.1, elevation in relation to tidal range and exposure to waves and currents are important abiotic factors governing salt-marsh formation. Salt marshes develop high in the intertidal zone in sheltered areas where fine-grained sediment can settle and accumulate (Allen and Pye 1992; Allen 2000). The mapping of high-potential areas for salt-marsh development therefore makes use of available information on (i) the bathymetry of the coastal zone, (ii) concentrations of fine-grained sediment in the upper sediment layer (as a proxy for lee conditions) and (iii) the velocity of currents along the coast (Table 2). The biotic classification is based on the presence of unique littoral and sublittoral habitats that provide important feeding areas for wading birds and spawning grounds for fish, sometimes also harbouring mussel (*Mytilus edulis*) banks (see *Wadden Sea Atlas*, http://documents.plant.wur.nl/imares/ecologische_atlas.pdf).

As presented in Fig. 5, besides long stretches where seminatural salt marshes are already present (some 73 km), there are several stretches along the Wadden Sea coast with favourable abiotic conditions for salt-marsh formation (some 15 km). However, such development may come at the cost of valuable littoral and sublittoral habitats. Furthermore, Fig. 5 presents stretches where salt-marsh development would require minimal effort (some 42 km) and considerable effort (some 56 km), in the form of raising the elevation via nourishments and possibly additional measures to prevent erosion and to cushion unfavourable conditions that cause increased local water depth or strong currents. Along some 75 km of dike, abiotic conditions are considered to be entirely unsuitable for salt-marsh development.

## Socioeconomic and governance aspects

### Use of Wadden Sea salt marshes

The Wadden region has a long tradition of beneficiary use of salt marshes. The first populations in the Wadden region settled on the natural high grounds in this tidal landscape. Salt marshes were used for hunting and fishing, as well as for grazing (cattle and sheep) and harvesting hay. Reclamation of salt marshes began in the Middle Ages, when artificial earth mounds were progressively connected by dikes, leading to the formation of dike rings protecting the hinterland (former salt marshes) (Cools 1948). When sedimentation on the seaward side of these dikes produced new salt marshes, new dikes were built to reclaim those areas for agriculture (both grazing and cultivation). Centuries of land reclamation caused the boundary between land and the Wadden Sea to gradually shift seawards, making natural salt-marsh formation increasingly difficult. Sedimentation was therefore actively stimulated from the seventeenth century onwards (Dijkema et al. 2001). This process of stimulating accretion and land reclamation continued until the twentieth century, when livestock grazing on the salt marshes became less rewarding economically. Since the 1970s, nature conservation organizations have acquired large stretches of salt marshes (e.g. Balgzand, Noorderleeg, Peazemerlannen, Noordpolderzijl, Punt van Reide and the eastern section of the Dutch Dollard salt marshes). On the Groningen mainland coast, however, extensive marsh areas are still owned by farmers and used for cattle grazing. Stemming from historical agreements, the Dutch Department of Public Works performs the necessary maintenance to protect these privately owned salt marshes against erosion. Moreover, the owners of the marshlands along this coast have initiated, in cooperation with local governmental bodies and nature conservation organizations, a project to improve conditions for cattle grazing (e.g. by creating animal refuge areas and maintaining the drainage ditches). In contrast to the privately owned marsh areas, the grazing regime on the salt marshes belonging to nature conservation organizations is geared more towards nature management purposes. Most salt marsh expanses on the Dutch Wadden islands are owned and managed by nature conservation organizations, although there are still some privately owned areas on the isles of Ameland and Terschelling.

### Conservation tasks and development goals

Increased interest in nature conservation starting in the 1970s led to incorporation of the entire Wadden Sea area, including the salt marshes, in the intergovernmental Ramsar Convention (officially, the Convention on Wetlands of International Importance, Especially as Waterfowl Habitat). The Wadden Sea is subject to various European Union (EU) directives as well, such as the Habitats Directive and the Wild Birds Directive (Council of the European Communities 1992). In order to implement these directives in national legislation, the Dutch Wadden Sea was brought under the national Nature Conservation Act and made a Natura 2000 area for which target species and protected habitats were articulated (Ministerie van Economische Zaken Landbouw en Innovatie 2011). Furthermore, in recognition of the area’s unique natural values, the United Nations Educational, Scientific and Cultural Organization (UNESCO) declared the Wadden Sea a World Heritage Site in 2009 (World Heritage Nomination Group 2008; UNESCO 2009). All of this means that the Dutch government is obliged to conserve the salt-marsh area in the Wadden Sea, including all succession stages and saltwater-to-freshwater transitions, as well as to monitor salt-marsh development (Ministerie van Economische Zaken Landbouw en Innovatie 2011). Interventions in the coastal zone that affect the Wadden Sea salt marshes (such as dike reinforcement) are bound by strict rules, and any loss of marsh area must be compensated.

Since 1960, the areal extent of the Dutch Wadden Sea salt marshes, their elevation and vegetation have been systematically monitored, including the effects of changes in the management of reclamation works since 1982 (Dijkema et al. 2013). The EU Water Framework Directive (2000/60/EC) sets additional requirements on salt-marsh extent as well as on vegetation structure, including a monitoring requirement. Thus, salt marsh vegetation cover has been monitored by aerial photographs since the implementation of this directive (see Esselink et al. 2009). To prevent erosion of the marsh areas along the mainland coast in Groningen and Fryslân, maintenance of the accretion works is prescribed. Beyond EU-level conservation efforts, there is the earlier-mentioned trilateral agreement between Denmark, Germany and the Netherlands, by which the signatories agree to increase the area of natural salt marshes, to enhance natural morphological and dynamic processes in the Wadden Sea region, to enrich the natural vegetation structure of artificial salt marshes and to improve conditions for wading birds (CWSS 1998; 2010). For these purposes, Dutch monitoring information is combined with Danish and German data for trilateral monitoring programmes.

### Management and maintenance

Historically, most salt marshes were used for livestock grazing. Grazing contributes to marsh biodiversity by slowing succession, preserving more species-rich succession stages and (depending on the livestock species and density) inducing patchiness of vegetation and structural heterogeneity (Adler et al. 2001; Nolte et al. 2014). This also influences ground-breeding birds and other animals. Without grazing, tall-growing species such as sea coach (*Elytrigia atherica*) outcompete shallow species in the higher elevation salt-marsh zones (Olff et al. 1997). However, livestock grazing on salt marshes must be monitored daily due to the risk associated with tidal inundation of the lower marsh zone, flooding during storm events and the silted up ditches that form a continuous danger of drowning for the animals. To reduce the risk of drowning, the ditches require regular maintenance. For these reasons, grazing on privately owned salt marshes has diminished since the 1970s. Grazing intensity has also been reduced in the areas owned by nature conservation organizations, as biodiversity and landscape objectives have changed (see e.g. Esselink et al. 2000). However, grazing in low stocking densities is a common means to preserve species-rich, seminatural grasslands. Recent experiments have sought to determine optimum grazing routines for maintenance of salt-marsh biodiversity (see e.g. Nolte et al. 2014).

As mentioned earlier, historically salt marsh forming was stimulated for land reclamation. However, maintenance of the salt marsh works (brushwood dams and sedimentation fields) is currently geared to nature conservation goals or forthcoming from historical agreements.

### Recreation and tourism

Recreation and tourism in the Wadden region is strongly linked to the area’s unique nature and landscape. Salt marshes and the adjacent mudflats are a prominent feature along the Wadden Sea coast. They offer interesting surroundings for walking, bird watching, nature excursions and mudflat hiking. Several Wadden salt marshes and summer polders have been made accessible by trail (e.g., Noorderleeg, Peazummerlannen, Noordpolderzijl) and bird hides have been constructed for visitors (e.g. at Dollard). Bicycle paths along long stretches of dike offer additional opportunities to enjoy the unique marsh landscape. Recently, a salt-marsh education centre was opened in Noorderleeg. This centre welcomed more than 10,000 visitors in its first year. Furthermore, each year, some 15,000 visitors join excursions organized by a local business to the Noorderleeg summer polder (pers. comm. K. Laansma).

Promoting appreciation of the Wadden landscape is a focus of the conservation programme ‘Towards a Rich Wadden Sea’ (Stuurgroep naar een Rijke Waddenzee 2010). In recognition of the contribution of salt marshes to landscape quality, this programme seeks ways to promote salt-marsh development at suitable locations.

## Discussion

At the start of the Wadden Programme in 2010, local stakeholders identified integration of vegetated forelands into flood defences as an interesting long-term adaptation strategy for further exploration. Combining salt-marsh development with improved flood protection was viewed as an appropriate way to meet the Wadden Programme’s objective to adapt to climate change while also strengthening the region’s nature and landscape values (Deltaprogramma Waddenzee 2011; 2012). The emerging interest in nature-based flood protection was strongly stimulated by the recommendations of the Second Delta Committee (Deltacommissie 2008), while also reflecting a global trend of reorienting coastal management towards a more flexible and adaptive approach and recognizing the contribution of natural processes to flood safety (e.g. King and Lester 1995; Costanza et al. 2008; Waterman 2008; Temmerman et al. 2013; Van Slobbe et al. 2013). Examples of this trend outside the Netherlands are the introduction of managed realignment (the deliberate breaching of existing sea defences resulting in the creation of salt marshes in the flooded land beyond the opened dikes) in the United Kingdom (UK) (Ledoux et al. 2005; French 2006; Turner et al. 2007); the inclusion of salt marshes in local sea defences and management schemes in Schleswig-Holstein (Germany) (Hofstede 2003) and the UK (Möller et al. 2001); maintenance and nourishment of existing marshlands and creation of new wetlands for coastal defence along, for example, the United States’ south-eastern coast (Feagin et al. 2010); and increasing interest in the buffering capacity of mangroves (see e.g. Ewel et al. 1998). Furthermore, the European Union has financed several integrated projects (e.g. COMCOAST, Coastview, Coast3D) that include theoretical, observational and experimental studies on this topic. With the introduction of the so-called natural climate buffer concept, multiple Dutch nature conservation organizations have expressed their interest in utilizing nature-based protection measures against the impacts of climate change as well (www.klimaatbuffers.nl). Even Dutch building and civil engineering companies have become involved with the Building with Nature initiative, which stimulates the use of nature and natural processes in development of hydraulic infrastructure (De Vriend and Van Koningsveld 2012).

From the start, representatives of the local water boards raised questions concerning the effectiveness of salt marshes for flood protection under extreme conditions. The Wadden Programme therefore focused on this topic, and commissioned several explorative modelling studies. The first explorative modelling results indicated that Wadden Sea salt marshes do attenuate wave heights, even under extreme conditions (Venema et al. 2012). A 600-m salt-marsh zone alongside all Wadden Sea dikes would, studies suggested, substantially reduce the need for dike reinforcement works up to 2050, if the salt marsh could keep pace with the rising sea level (Calderon and Smale 2013). Notwithstanding these promising modelling results, the water boards continued to stress the lack of field data on the effects of natural vegetated foreshores on wave attenuation during extreme storm events. Wave models are developed and evaluated on the basis of either scaled down lab tests or field measurements under less than extreme conditions. Hence, applying these models to predict effects under extreme conditions relies on extrapolation. Unfortunately, the Delta Programme’s funding did not allow field measurement or flume studies to verify whether wave attenuation was accurately quantified by the applied model.

Although the Wadden Programme was unable to convince all of the involved stakeholders to fully accept the concept, they did ultimately define integration of salt marshes into flood defences as one of the preferred adaption strategies to explore for the Wadden region (Gerritsen et al. 2014). The concept has now been included in a major research programme under the umbrella of a new national Dutch flood protection programme. Furthermore, as a follow-up to the Wadden Programme’s efforts, other research programmes are getting under way and will seek to further evaluate and calibrate the existing models, to reduce prediction uncertainties. Monitoring of wave attenuation along the Wadden Sea coast and to compare modelled wave heights with observed heights or debris marks on dikes after storms has already begun.

The Wadden Programme’s literature studies served a vital role in compiling and disseminating an abundance of scientific knowledge and experience to the stakeholders involved. Much of this information had been available only in scattered form, in both scientific and grey literature. This facilitated communication between the Wadden Programme and local stakeholders. This common starting point enabled a broad range of stakeholders to participate in the process of identifying promising and preferred climate adaption strategies for the Wadden region. These literature studies did not, however, diminish the concerns of some Wadden Sea experts regarding possible trade-offs for nature and biodiversity. These experts felt bound to the goal of salt-marsh conservation as laid out in various national and international agreements. Some of them had been involved in monitoring programmes for decades and taken part in numerous studies on best practices in salt-marsh management for the purpose of biodiversity conservation. Trade-offs arise from the fact that flood protection is required mainly during extreme events, which impose rather different requirements on the extent and features of salt marshes than biodiversity conservation (Van Loon-Steensma and Vellinga 2013). To be effective during storm conditions, the salt-marsh surface needs to be relatively high, whereas a relatively low-lying and dynamic salt marsh is best for biodiversity. Furthermore, the flood protection service is better assured by a stable salt marsh. This often requires erosion protection, which reduces the salt marsh naturalness. Although the salt-marsh potential map presented in this paper provides an impression of promising locations for integrating salt marshes into flood defences, it would be wise to involve more salt-marsh experts in identification of pilot locations and in implementation of any pilot projects. In addition to providing an opportunity to utilize all available expertise, this could contribute to finding solutions with greater synergy (and minimal trade-offs), and it may also help to overcome resistance.

Because wave damping is determined largely by the breaking of waves (which occurs in the first metres of the salt marsh), a narrow salt-marsh zone may be sufficient for effective flood protection. However, salt marshes are dynamic. There is still little experience in quantifying and assessing their dynamism and in integrating them into dike designs. Therefore, to guarantee wave damping during extreme events, it would be wise to include some over-dimensioning of the required foreshore width. Such over-dimensioning would also present opportunities for combining functions in the foreshore, because over-dimensioning of a vegetated foreshore allows flexibility for natural processes, as well as spaces for recreation or agricultural uses. The resulting robust, multifunctional flood defence zone would represent a combined approach to coastal climate change adaptation, which is considered prudent in a highly uncertain environment (e.g. Moser et al. 2012; Cheong et al. 2013).

Finally, the prospects for integrating salt marshes into flood defences as an adaptation strategy for the Wadden region also depends on aspects other than the main physical factors governing salt-marsh dynamics (such as elevation of the nearshore zone, sediment supply, wind-wave dynamics and sea level). Various nature conservation agreements are in effect with their associated obligations. Prospects will likely hinge on the foreseen value of salt marsh development compared to traditional reinforcements, in terms of both costs and benefits. Although ecosystem creation and restoration are often advocated as cost-effective (see e.g. Temmerman et al. 2013), there is still too little quantitative information to be certain of this. Reasonably better understood are the benefits related to flood protection, which can be measured by the avoided costs of dike reinforcement (see e.g. King and Lester 1995; Dixon et al. 1998) and damages avoided (see e.g. Costanza et al. 2008). Furthermore, ecological and landscape qualities that may subsequently influence recreation and tourism are not well understood and are difficult to quantify. Among the costs incurred are those concerning preservation, development and maintenance of salt marshes, as well as changes in estuarine habitats, which are difficult to monetize. The ecosystem-service concept may proof a useful concept to value all costs and benefits involved in the integration of salt marshes in a long-term adaptation strategy for the Wadden region and to underpin future decision making (de Groot 2006; de Groot et al. 2010; Luisetti et al. 2011).
